# Density of the midpalatal suture after RME treatment – a retrospective comparative low-dose CT-study

**DOI:** 10.1186/1746-160X-10-18

**Published:** 2014-05-20

**Authors:** Michael Schauseil, Björn Ludwig, Berna Zorkun, Andreas Hellak, Heike Korbmacher-Steiner

**Affiliations:** 1 University of Marburg, Georg-Voigt-Str. 3, Marburg 35039, Germany; 2 Orthodontic praxis in Traben-Trarbach, Traben-Trarbach 56841, Germany; 3 Trakya University, Edirne 22030, Turkey

## Abstract

**Introduction:**

Rapid maxillary expansion (RME) is a common technique to improve the dental and skeletal transverse width in cases of constricted maxillary arches. Although retention after RME has been widely examined, there is still no clear statement about the minimal retention time in postpubertal patients and many practitioners have retention concepts varying between three and six months.

**Methods:**

This retrospective study consisted of 14 patients who were either treated with a Haas-type RME (6 patients) or a Hybrid-RME (8 patients). The average age was 15.8 years (min. 13.5 years, max. 23.0 years).

Low-dose CT scans were taken initially before placement of the RME (T0), directly after maximal activation (T1) and (in six cases) also in retention after 6 months (T2). Using a 3D-software (“OnDemand3D”/Cybermed Inc.) in analogy to the method published by Franchi et al. (*AJODO Volume 137*/ *Number 4*) all values were measured twice at an interval of 1 month to assess the method error and the intraoperator reliability.

Statistical analysis was performed using SPSS 21 for Mac. Possible influences of the RME-type were assessed using the univariate ANOVA. Changes in the sutural density between the different points of time were examined using paired t-tests.

**Results:**

The density of the suture decreased significantly after expansion (T0-T1) with both types of RME (p = 0.000). In the retention period there was a significant increase of the sutural density (p = 0.007) although it did not achieve the initial level (p = 0.002).

**Conclusions:**

1. The midpalatal suture was opened in all analysed patients.

2. In postpubertal patients a retention time of six months does not allow sufficient reorganization of the suture.

3. Therefore, a retention period longer than six months seems to be beneficial to prevent relapses in postpubertal patients.

## Introduction

Rapid maxillary expansion (RME) is a common technique to improve the transverse dimension in patients with constricted maxillary arches [1]. Due to animal experiments on RME, it is known that the suture opening is histologically characterized by stretched fibrous conjunctive tissue and massive invasion of osteoclast within the inner suture [2]. During the retention period the tissue becomes reorganized and in terms of the histological appearance the suture shows no differences in comparison to the initial suture before RME. According to systematic reviews regarding long-term stability of the expansion, only approximately 25% of the initial achieved widening remains [3]. Therefore it is essential to overcompensate the active expansion [4] and to stabilise the result with a sufficient duration of the retention period [5].

Since the procedure of RME after the pubertal peak tends to show more relapses [6], it is important to plan a sufficient retention period with older patients. Although retention after RME has been widely examined [3,5,7], there is still no clear statement about the minimal retention time in postpubertal patients. Usually the retention period varies between 3 and 6 months [8-10].

With the help of computed tomography (CT) the dimensional changes of the suture during RME were documented in recent clinical studies. The evaluation of Hounsfield index has been successfully used in order to assess the bone density in possible implant sites. In analogy Franchi et al. examined the sutural remodellation after RME in a Low-dose CT-study with the help of Hounsfield measurements [9]. Their prepubertal patients had an average age of 11.2 years, and prepubertal stages of cervical vertebral maturation (C1-C3). They stated that after a retention time of 6 months, the midpalatal suture was reorganized showing a density similar to the pre-treatment values [9].

To our knowledge, no study exists so far analysing the sutural remodellation after RME in adolescents after pubertal peak (CVM >3). In their discussion Franchi et al. demanded a future study dealing with questions of clinical relevance in older patients. Therefore, the aim of our study was to examine the sutural changes in postpubertal patients after RME and during the retention period.

Hybrid-RME treatment was introduced by Wilmes [11,12] to establish a more direct transfer of the Hyrax force to the hard palate. This aspect is especially interesting in older patients, since age-related changes of the suture may require more expansive force on the suture. However there is no study published until now proving this hypothesis.

Our second aim was to examine if Hybrid-RME is able to open the midpalatal suture in patients after the pubertal peak with a CVM-stage larger than three.

## Materials and methods

This retrospective study comprised 14 patients (4 boys, 8 girls) who were all treated by the same examiner (B.Z.). The average age was 15.8 years (min. 13.5 years, max. 23.0 years). Inclusion criterion was a skeletal maturation level not lower than four.

**Figure 1:**
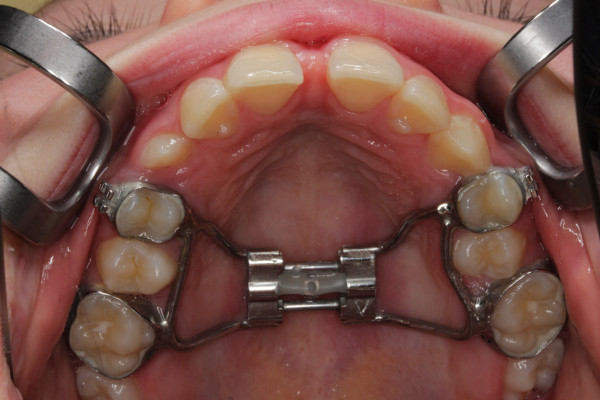
Conventional RME was used in 6 patients.

**Figure 2:**
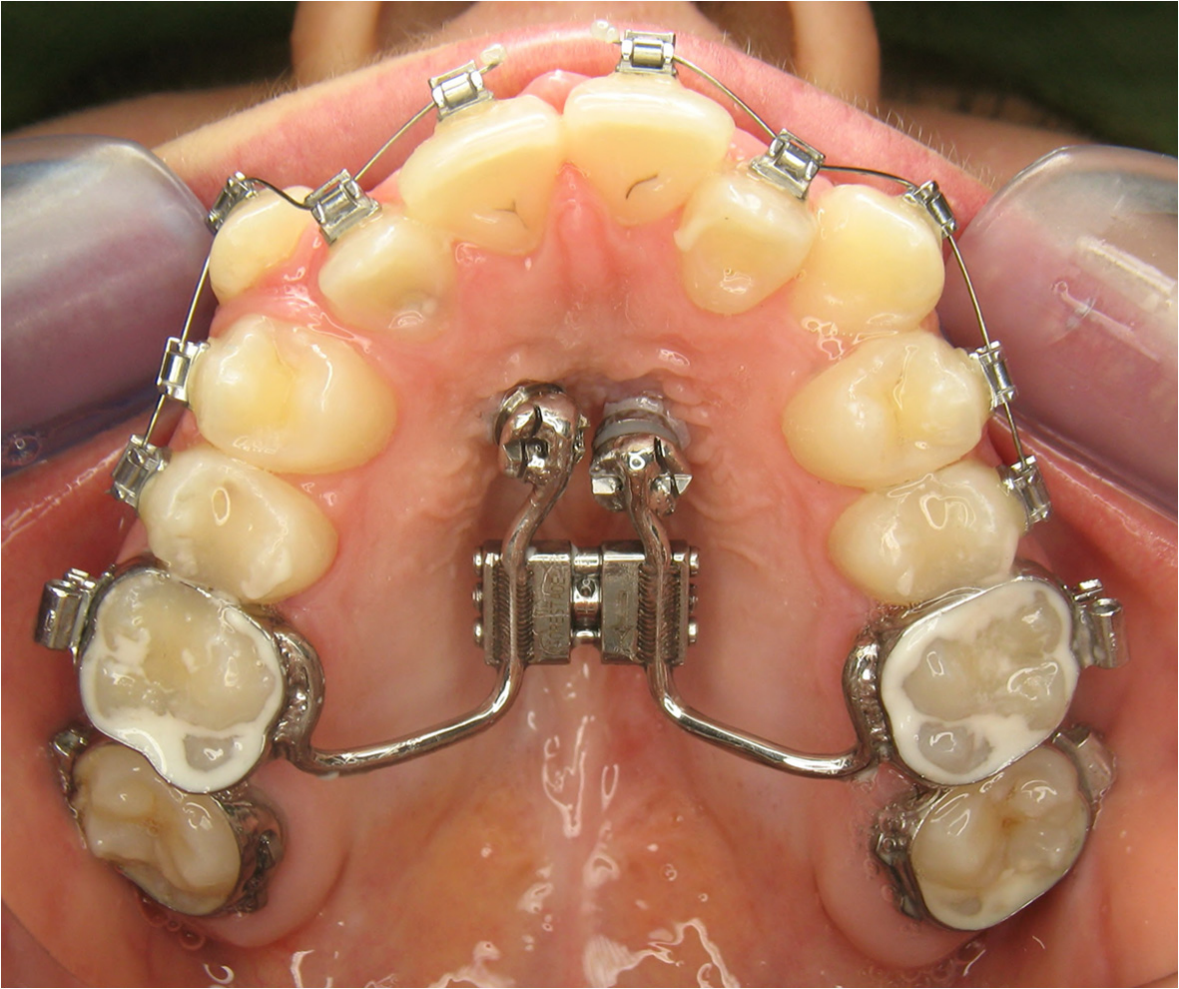
Hybrid-RME (TAD’s in the anterior palate combined with molarbands) was used in 8 patients.

All patients had a nasomaxillary constriction and were treated either with a Haas-type RME (6 patients, Figure 1) or a Hybrid-RME (8 patients, Figure 2). The decision to implement skeletal anchorage was made with regards to the amount of transverse expansion needed, and the local anatomic conditions.

Each patient was instructed to activate the Hyrax-screw (0.25 mm per turn) three times a day.

The retrospective examination of the Low-dose CT’s was reviewed and approved by the ethics commission of Saarland (Saarbruecken, Germany) with the approval number 170/12.

**Figure 3:**
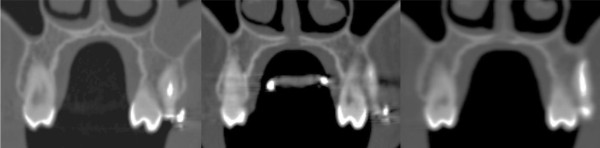
Representative coronal slices: left: before RME (T0), middle: after maximal expansion (T1) and right: after 6 months of retention (T2).

**Figure 4:**
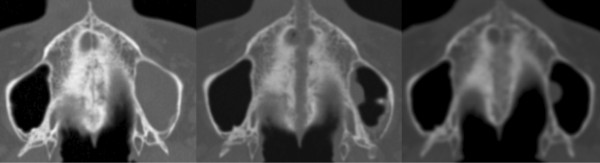
Representative axial slices: left: before RME (T0), middle: after maximal expansion (T1) and right: after 6 months of retention (T2).

Low-dose CT (Phillips, Brilliance CT 16) scans were taken initially before placement of the RME (T0), directly after maximal activation (T1) and (in six cases) also in retention after 6 months (T2). The analyses of the data sets were performed blinded using a free trial of the software “OnDemand3D” (Cybermed Inc., Irvine, CA, USA) (Figures 3 and 4) [13].

**Figure 5:**
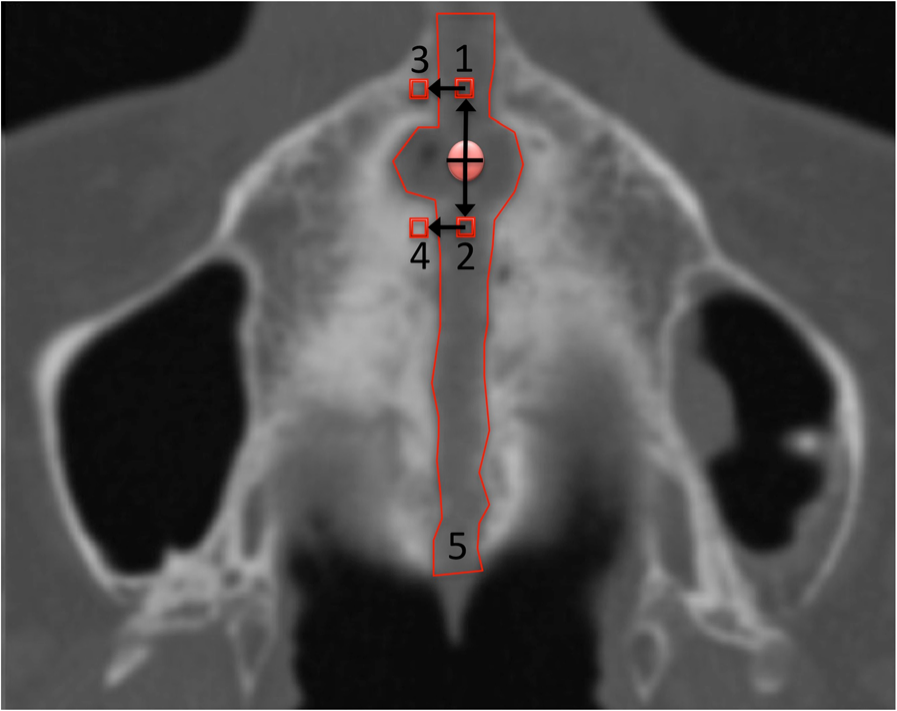
**Illustration of measured areas as published by Franchi et al.** 1. Anterior sutural ROI (AS ROI): density values measured in the ROI located along the midpalatal suture 5 mm in front of the center of the nasopalatine duct at T0, T1, and T2. 2. Posterior sutural ROI (PS ROI): density values measured in the ROI located along midpalatal suture 5 mm posterior to the center of the nasopalatine duct at T0, T1, and T2. 3. Anterior bony ROI (AB ROI): density values measured in the ROI located on the palatal bone 3 mm laterally (on the right side) to the AS ROI at T0. 4. Posterior bony ROI (PB ROI): density values measured in the ROI located on the palatal bone 3 mm laterally (on the right side) to the PS ROI at T0. 5. Additionally to the rectangular ROI values “polyline”-measurements of the entire suture were performed in regard to the individual anatomy.

All 34 CT-scans were evaluated by the same trained operator (M.S.) using the same computer (2,8 GHz Intel Core i7, Windows 7). Axial CT-Scans were calibrated in analogy to the method published by Franchi et al. [9] and the Hounsfield density in the midpalatal suture was measured at 4 defined Region-of-Interest (1 mm^2^) as proposed by Franchi et al. (Figure 5).

In addition to the rectangular ROI values “polyline”-measurements of the entire suture (visible on the axial slices) were performed at T0, T1 and T2 in regard to the individual anatomy.

All values were measured twice at an interval of one month to assess the method error and the intraoperator reliability.

### Statistical analysis

Statistical analysis was performed using SPSS 21 for Mac. Normal distribution was tested with Shapiro–Wilk test and graphic data output. The intraoperator reliability was measured using paired t-tests and the Pearson correlation coefficient. Possible influences of the RME-type were assessed using the univariate ANOVA. Changes in the sutural density between the different points of time were examined using paired t-tests. The differences between the overall sutural density-changes at all 3 points of time were named “initial decrease” and “regeneration”, respectively. The significance level was set to 0.05.

## Results

**Figure 6:**
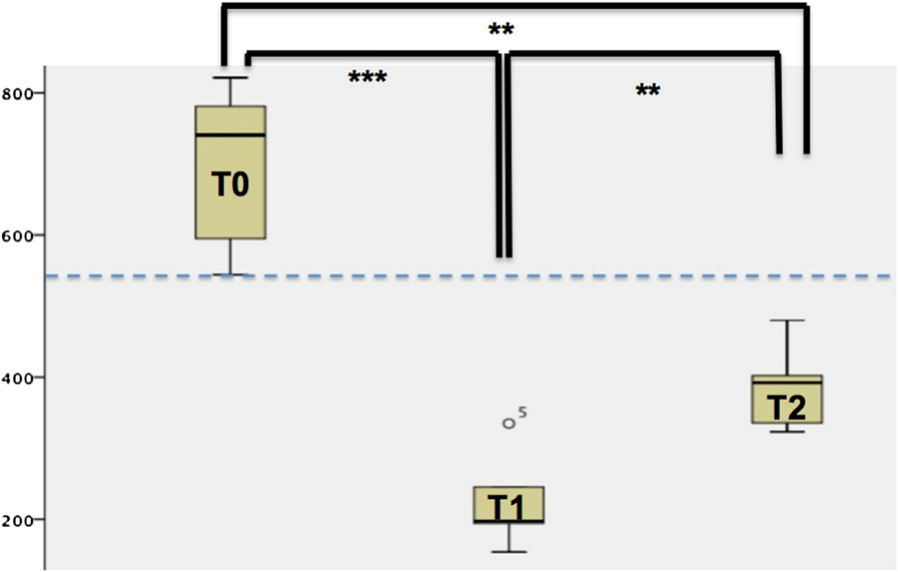
**Density changes during RME**-**treatment.***y*-*axis*: Density-level in Hounsfield-units (HU). *x*-*axis*: The density decreased significantly after expansion (T0-T1) (p = 0.000). In the retention period there was a significant increase of the sutural density (p = 0.007) although it did not achieve the initial level (p = 0.002).

Paired t-tests and Pearson coefficient showed a high intraoperator correlation (>0.9; p = 0.000). As demonstrated in Figure 6, the density of the suture decreased significantly both in the anterior and the posterior region of the suture after expansion (T0-T1) with both types of RME (p = 0.000). This effect was also seen in the analysis of the entire suture (p = 0.000). In the retention period, there was a significant increase of the sutural density (p = 0.007) although it did not achieve the initial level (p = 0.002).

There was no significant difference between the density of AS-ROI’s and PS-ROI’s in regard to the ROI’s 3 mm laterally (p = 0.127).

The patients treated with Hybrid-RME were older than those treated with conventional RME (17.2 vs. 13.9 years) and had a significantly higher maturity of the midpalatal suture at the beginning (p = 0.019). Because Hybrid-RME induced a higher decrease of the density (469 + -82 HU) than the conventional RME (365 + -88 HU) (p = 0.041), the density of the midpalatal suture after maximal expansion was not significantly different between both groups at T1 (p = 0.4).

## Discussion

Both Lione et al. [10] and Franchi et al. [9] proposed a retention time of six months after sutural expansion to be sufficient for prepubertal patients (CVM 1–3). In their Low-Dose-CT study both authors examined 17 patients with an average age of 11.2 years (8–14 years) and demanded a future investigation of a comparatively older cohort group [9].

Chronological age cannot recognize the onset of the adolescent peak in skeletal maturation [14]. Because total length increments reach their maximum between CVM stages two and three [15], only patients with a cervical vertebral maturation level of not lower than 4 were involved in this study.

In adult cases transverse discrepancies can often hardly be resolved using conventional RME without previous surgical weakening. Because Hybrid-RME establishes a direct skeletal force, young adult patients can have a better chance for suture opening even without surgical assistance. In any case, before the insertion of RME all patients should be informed that surgical assistance might be necessary if the suture does not open. However, in this study group all RME procedures were carried out successfully without a need for surgical assistance.

In contrast to the results of Franchi et al. no significant differences between the midsutural region and the paramedial area (3 mm beside the suture) were detected. This can be explained by the age-related progressed ossification along the midpalatal suture.

In this study, the Hounsfield-value were not only analysed in a rectangular ROI but also in the entire suture as an additional benefit of the polyline-feature. The method error was equally low so this may be a useful method in addition to the application of Franchi et al. to analyse the whole suture in a reliable way.

The Low-Dose-CTs were taken at three different points of time: initially (T0), after maximal expansion (T1) and after six months of retention (T2). The sutural density was significantly smaller after RME, no matter if we focused on the anterior, the posterior or the overall sutural region. After six months of retention there was a significant increase of the sutural density.

In their younger cohort, Franchi et al. showed that there was no statistically significant difference between the sutural density at the beginning (T0) and after six months of retention (T2). On the contrary in our study the examined older group showed a slower regeneration process characterized by significant lower values at T2 in comparison to T0; this applied to all cases.

The changes of density after expansion indicate that in older patients Hybrid-RME may induce a decrease of sutural density to a level comparable to younger patients treated with conventional RME. Therefore, Hybrid-RME can open the suture even in cases of older patients and thus might be advantageous especially for their sutural expansion. Nevertheless, it must be pointed out that in this study we only had six patients with Low-Dose-CT at T2. The statistics would be more significant if we had a greater number of patients’ CT at this point of time. Since this study had a retrospective design, it was not possible to add those T2 records for additional patients. In order to deal with the issue of small sample sizes, it would be more reliable to observe cohort groups in retrospect, but any new CT study should be avoided to prevent the radiation effect.

## Conclusions

1. The midpalatal suture was opened in all analysed patients.

2. In postpubertal patients a retention time of six months does not allow sufficient reorganization of the suture.

3. Therefore, a retention period longer than six months seems to be beneficial to prevent relapses in postpubertal patients.

## Competing interests

The authors declare that they have no competing interests.

## Authors’ contributions

MS carried out the measurements, the statistics and drafted the first manuscript. BL, BZ and AH helped with the design, coordination of the study and the draft of the manuscript. HKS helped with the study design, the translation into English language and the illustrations. All authors read and approved the final manuscript.
